# Anticonvulsant Potential of 1-Aryl-6,7-dimethoxy-1,2,3,4-tetrahydroisoquinolines: Insights from Strychnine and Nicotine Models in In Vivo and In Silico Studies

**DOI:** 10.3390/ph18091350

**Published:** 2025-09-09

**Authors:** Azizbek A. Azamatov, Nilufar Z. Mamadalieva, Asmaa A. Mandour, Sherzod N. Zhurakulov, Urkhiya K. Aytmuratova, Valentina I. Vinogradova, Fazliddin S. Jalilov, Firuza M. Tursunkhodzhaeva

**Affiliations:** 1 Institute of the Chemistry of Plant Substances, Uzbekistan Academy of Sciences, M. Ulugbek Str 77, Tashkent 100170, Uzbekistan; azizbek.azamatov@bk.ru (A.A.A.); n.mamadaliyeva@afu.uz (N.Z.M.); j.sherzod.78@mail.ru (S.N.Z.); urxiyaaytmuratova@mail.ru (U.K.A.); cnc@icps.org.uz (V.I.V.); 2 Faculty of Pharmacy and Chemistry, Alfraganus University, Yuqori Qoraqamish Str. 2a, Tashkent 100190, Uzbekistan; f.jalilov@afu.uz; 3 Pharmaceutical Chemistry Department, Faculty of Pharmacy, Future University in Egypt, Cairo 11835, Egypt

**Keywords:** 1-aryl-1,2,3,4-tetrahydroisoquinoline, anticonvulsant, seizure, nicotine, strychnine, molecular modeling, ADMET

## Abstract

**Background:** Epilepsy is a chronic, non-communicable brain disorder characterized by recurrent seizures. Some derivatives of 1,2,3,4-tetrahydroisoquinolines have demonstrated anticonvulsant effects. This study aims to investigate the effects of 33 derivatives of 1-aryl-1,2,3,4-tetrahydroisoquinoline on seizures induced by nicotine and strychnine. **Methods**: The anticonvulsant effects of 1-aryl-1,2,3,4-tetrahydroisoquinoline derivatives were evaluated in white male mice. Convulsant agents were administered subcutaneously at doses of 10.0 mg/kg for nicotine and 1.5 mg/kg for strychnine, 60 min after the oral administration of the test compounds at doses ranging from 0.1 to 10 mg/kg. The onset time, duration of tremors and seizures, and survival rate of the animals were recorded. The docking studies were conducted for 32 tested compounds targeting the α-amino-3-hydroxy-5-methyl-4-isoxazolepropionic acid (AMPA) receptor (PDB ID: 1FTL). Furthermore, a predictive ADMET study was conducted to evaluate the pharmacokinetic and toxicity profiles of the compounds. **Results:** Compounds **20** and **25** exhibited the highest activity against strychnine-induced seizures. When evaluating the effects of 1-aryl-1,2,3,4-tetrahydroisoquinolines and reference drugs on the tremorogenic and convulsive actions of nicotine at doses of 0.1–5 mg/kg, compounds **3**, **6**, **8**, **14**, **16**, **25**, **27**, **29**, **30**, **31**, and **34** demonstrated comparable activity to the reference drugs. The docking results targeting AMPA (PDB ID: 1FTL) revealed comparable binding interactions for most of the compounds, with a (−)C-Docker interaction energy range of 33.82–45.41 Kcal/mol, compared to that of the ligand (41.60 Kcal/mol). The structural requirements of the studied scaffold were analyzed to identify the essential pharmacophoric features for anticonvulsant activity. Furthermore, a predictive ADMET study was conducted to evaluate the pharmacokinetic and toxicity profiles of the compounds. **Conclusions**: Certain derivatives of 1,2,3,4-tetrahydroisoquinolines may serve as potential anticonvulsant agents for epilepsy.

## 1. Introduction

Epilepsy is a serious neurological disorder including spontaneous convulsions that might be accompanied by loss of consciousness. The abnormal electrical seizures appearing as episodic impulses are caused by brain neurons and are mainly related to imbalance between excitatory and inhibitory synaptic processes [1]. Neurotransmitters that show specific roles in neuronal processes related to epilepsy include gamma-aminobutyric acid (GABA), norepinephrine, and excitatory amino acids, such as glutamate and aspartate. Some antiepileptic treatments act by blocking Ca^2+^ channels, leading to prolonged Na^+^ channel inactivation, or by enhancing inhibitory GABAergic neurotransmission. Other, newer anticonvulsants target antagonism to glutamatergic neurotransmission [1,2,3].

Glutamate is the major stimulatory neurotransmitter in the central nervous system with ionotropic (iGluRs) and metabotropic (mGluRs) receptors. The large group of iGluRs includes an important therapeutic targeted subfamily, known as α-amino-3-hydroxy-5-methyl-4-isoxazolepropionic acid receptors (AMPARs), with specific pharmacological and functional properties in CNS neurotransmission, revealing a crucial role in synaptic plasticity as well, which is highly correlated to learning functions and memory. It was observed that AMPAR over-activation leads to neuronal cell death in different neurological diseases such as stroke, epilepsy, etc. These investigations showed that AMPAR antagonists could be considered as appealing therapeutic candidates for these disorders [4,5,6]. AMPA glutamate receptors (AMPARs) have essential features existing in multiple available sites for ligand interaction as receptor modulators. A competitive agonist or antagonist mechanism of action was well determined via X-ray diffraction. On the other side, AMPAR non-competitive ligands were evaluated based on receptor similarity to the allosteric binding site of NMDA antagonists. Recently, AMPAR non-competitive ligands have been developed for their independent effect on glutamate level and their advantageous lack of normal glutamatergic activity disturbance after prolonged use [4,5,6].

The convulsant activity produced by strychnine is associated with postsynaptic inhibition mediated by the amino acid glycine, which acts as an inhibitory neurotransmitter for motor neurons and interneurons in the spinal cord. Strychnine acts as a selective potent antagonist, blocking the inhibitory effects of glycine on glycine receptors [7]. Additionally, strychnine interacts with the agonist binding site of the nicotinic acetylcholine receptor in chromaffin cells, exerting a pharmacological effect independently of the glycine receptor [8]. It was reported that cholinergic signaling plays an important role in the development of epilepsy. Clinical data showed that in the hippocampus of patients with seizures, there is reduced binding of the mAChR (muscarinic acetylcholine receptors) antagonist I-iododexetimide, indicating a disruption of cholinergic transmission in epilepsy [9]. It was reported that there is involvement of nAChRs (nicotinic acetylcholine receptors) in the pathogenesis of experimental and clinical forms of epilepsy. Moreover, nAChRs are considered as regulators of Na^+^-dependent excitatory neurotransmission [10,11,12].

**Figure 1:**
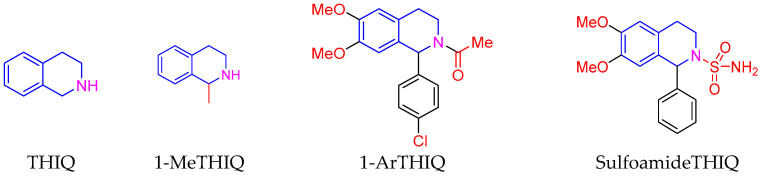
Tetrahydroisoquinoline and some of its derivatives.

Some derivatives of 1,2,3,4-tetrahydroisoquinolines showed anticonvulsant effects (Figure 1). One of them is 1-methyl-1,2,3,4-tetrahydroisoquinoline (1-MeTHIQ), which was known for Parkinson’s disease treatment; it exhibits anticonvulsant activity by increasing the threshold for electroconvulsions and potentiating the anticonvulsant effects of carbamazepine and valproate during maximal electroshock [13]. Activation of N-methyl-D-aspartate (NMDA) receptors induces a pattern of burst firing in neurons reminiscent of the paroxysmal depolarizing shifts (PDS) recorded in the epileptic brain [14]. AMPA (α-amino-3-hydroxy-5-methyl-4-isoxazolepropionic acid) receptor antagonists have a broad spectrum of anticonvulsant activity [15]. A three-dimensional pharmacophore model predicting anticonvulsant activity against audiogenic seizures in DBA mice was developed for 17 derivatives of 6,7-dimethoxy-1,2,3,4-tetrahydroisoquinoline as a new class of non-competitive AMPA receptor antagonists [16,17]. The best statistical hypothesis, created using the HypoGen module of Catalyst 4.9, consisted of five features: two hydrogen bond acceptors, two hydrophobic features, and one hydrophobic aromatic region, resulting in a model with a correlation coefficient of 0.919.

**Scheme 1:**
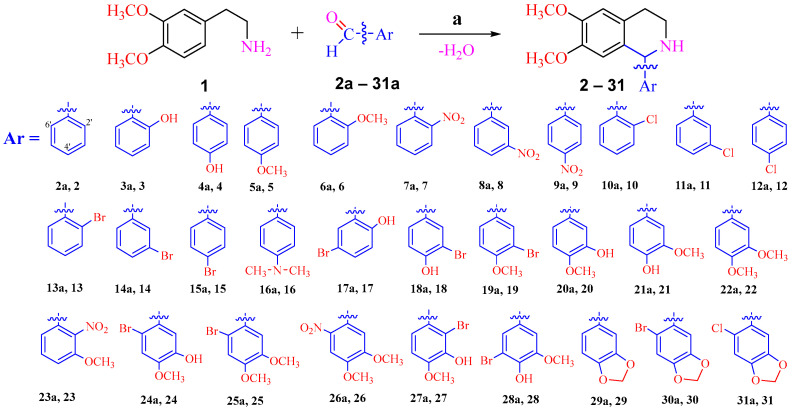
Synthesis of 1-aryl-6,7-dimethoxy-1,2,3,4-tetrahydroisoquinoline derivatives **2–31** (a: CF_3_COOH, reflux, 4–6 h).

**Scheme 2:**
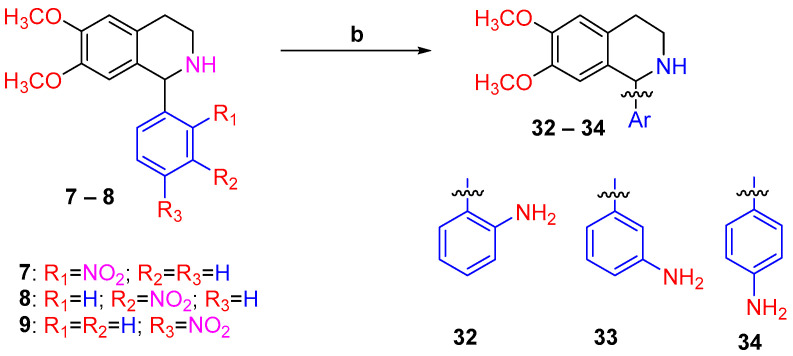
Reduction of 1-(2′-nitrophenyl) (**7**), 1-(3′-nitrophenyl) (**8**), and 1-(3′-nitrophenyl)-6,7-dimethoxy-1,2,3,4-tetrahydroisoquinolines (**9**), resulting in the formation of derivatives **32–34** (b: SnCl_2_×2H_2_O, HCl, C_2_H_5_OH, reflux, 4 h).

In a previous study [18], we investigated the toxicity of 20 derivatives of 1-aryl-6,7-dimethoxy-1,2,3,4-tetrahydroisoquinolines, analyzed the structure–toxicity relationship, and identified compounds with local anesthetic activity. Several 1-aryl-6,7-dimethoxy-1,2,3,4-tetrahydroisoquinoline derivatives displayed potent anticonvulsant effects in different animal models of epilepsy [17,19]. The presented research aims to investigate the anticonvulsant effects of thirty-three derivatives of 1-aryl-1,2,3,4-tetrahydroisoquinoline (Scheme 1 and Scheme 2) on seizures induced by nicotine and strychnine. These studies are essential for predicting the anticonvulsant effects and potential side effects of the examined derivatives, which may serve as a basis for the development of new antiepileptic drugs. Additionally, in silico studies were conducted, applying molecular docking simulation to investigate the binding mode of the tested compounds at the binding site of α-amino-3-hydroxyl-5-methyl-4-isoxazole propionic acid (AMPA) (PDB ID: 1FTL), via calculating the binding energy and visualizing the binding orientations within the active site compared to the ligand. The structure–activity relationship of the compounds was studied to determine the essential structural features required for their anticonvulsant activity. Furthermore, an in silico ADMET study was demonstrated to determine the drug-likeness of the tested compounds and predict their toxicity level.

## 2. Results

### 2.1. Synthesis of the 1-Aryl-1,2,3,4-tetrahydroisoquinoline Derivatives

Derivatives of 1-aryl-1,2,3,4-tetrahydroisoquinoline **2–31** were obtained as described in [18].

### 2.2. Pharmacological Evaluation

The structures of some derivatives of the studied scaffold were preliminarily evaluated for prediction of biological activity using the PASS (Prediction of Activity Spectra for Substances) online web resource (Version 2.0). Many investigated derivatives were predicted with high probability to have nicotinic receptor agonistic or antagonistic activity. This prediction as well as data previously published by other scientists and provided in the introduction were the impetus for our investigations.

#### 2.2.1. Strychnine Seizure Model

The animal study of the effects of 1-aryl-1,2,3,4-tetrahydroisoquinolines on the convulsive action of strychnine (Table 1) showed that in the control group, 100% of the animals died, with seizures occurring on average after 9.8 min. The average duration of seizures was 0.52 min. Carbamazepine increased the survival rate of the animals by 40–50%, while Convulex increased it by 20–40%. Carbamazepine at doses of 5–10 mg/kg increased the latency time for seizure onset by 29–49% and reduced the duration of seizures by 21–37%. In contrast, Convulex at doses of 75–100 mg/kg increased the latency time by 46–57% and reduced the duration of seizures by 53%.

All studied 1-aryl-6,7-dimethoxy-1,2,3,4-tetrahydroisoquinolines reduced animal mortality by 20–90%, depending on the administered dose. An optimal dose for maximal efficacy was 1.0 mg/kg. Compounds **20** and **25** were the most active against strychnine-induced seizures. Compound **20**, at doses of 1–10 mg/kg, increased animal survival during strychnine-induced seizures by 90%, while extending the latency period for seizures by 27–86% and having minimal effect on the duration of the seizures. Compound **25**, at doses of 0.5–1 mg/kg, increased animal survival by 90%, extended the latency period for seizures by 30–100%, and reduced their duration by 3.7–4 times. Thus, both compounds demonstrated greater anticonvulsant activity compared to the reference drugs in the strychnine-induced seizure model. The acute toxicity of these compounds when administered orally is 980 mg/kg (**20**) and 290 mg/kg (**25**) [18].

#### 2.2.2. Nicotine Seizure Model

The results of the study on the compounds effects in the nicotine-induced seizure model are presented in Table 2. In the nicotine seizure model, the mortality rate of the control group was 70%. In this group, nicotine at a dose of 10 mg/kg caused tremors and seizures within 40 s after administration, with a duration of 13.5 ± 0.8 min. When studying the effects of 1-aryl-1,2,3,4-tetrahydroisoquinolines and reference drugs on the tremorogenic and convulsive actions of nicotine at doses of 0.1–5 mg/kg, the compounds **3**, **6**, **8**, **14**, **16**, **25**, **27**, **29**, **30**, **31**, and **34** showed comparable activity to the reference drugs. These compounds, at low doses, increased the survival rate of the animals to 90–100%. For carbamazepine, this rate was 60–80%, and for valproate, it was 90%. The acute toxicity (LD_50_) of the most active derivatives of 1,2,3,4-tetrahydroisoquinoline (1,2,3,4-TGIQs) increases in the order: **27** < **18** < **34** < **30** < **31** < **8** < **16** < **14** < **3** < **6** < **25** < **29** [18]. The results indicate that compound **27**, with an LD_50_ of 4937.4 mg/kg, and **18**, with an LD_50_ of 3793.8 mg/kg, exhibit the lowest toxicity combined with high anticonvulsant activity in the nicotine seizure model. These compounds possess both a bromine atom and a hydroxyl group at C-2′ and C-3′, contributing to a survival rate of 90% in animals starting from a dose of 0.5 mg/kg. Compound **6** demonstrates the highest anticonvulsant activity, leading to a survival rate of 90% in nicotine-poisoned animals from a dose of 0.1 mg/kg.

### 2.3. Molecular Docking

**Figure 2:**
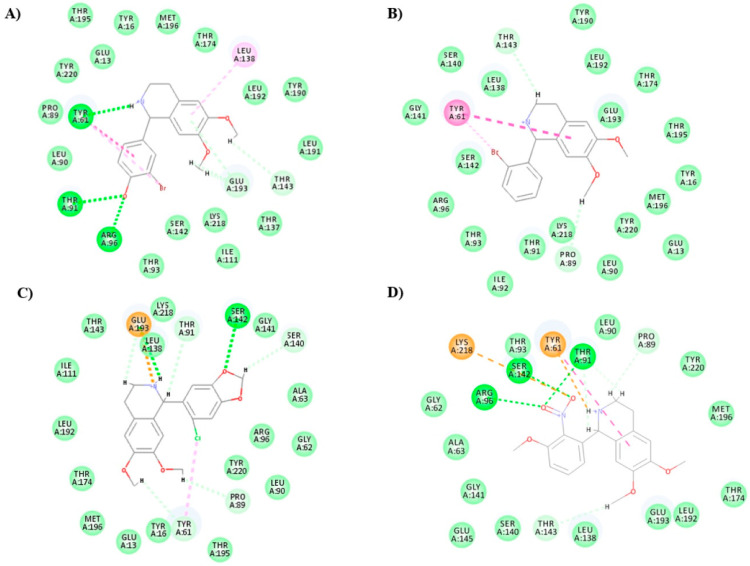
The binding interactions with amino acid residues in the active site of AMPA (PDB ID: 1FTL). (**A**): Compound **18** (E = −45.41 kcal/mol), (**B**): compound **13** (E = −33.82 kcal/mol), (**C**): compound **31** (E = −40.36 kcal/mol), (**D**): compound **23** (E = −44.10 kcal/mol)(Color code for interactions: H-bond: Green, carbon-H bond: light blue, Pi-Sigma bond: blue, Pi-Pi bonding: purple, Pi-alkyl bond: Pink, Pi-carbon & attractive charge: Orange).

The docking results of the 32 tested compounds targeting α-amino-3-hydroxyl-5-methyl-4-isoxazole propionic acid (AMPA) (PDB ID: 1FTL) are presented in Table 3. The 2D interactions were compared to that of the downloaded ligand after re-docking with RMSD value = 0.5A°. All the tested compounds showed a comparable binding mode, with (−) C-Docker interaction energy range E = −33.82–45.41 Kcal/mol, compared to the ligand (E = −41.60 Kcal/mol). By visualization, it was revealed that the ligand formed five hydrogen bonds, including two hydrogen bond acceptors (HBAs) with ARG96 and three HBAs with each of THR174, THR195, and GLU193, as well as one hydrogen bond donor (HBD) with PRO89 and one carbon–hydrogen bond with LEU90. Hydrophobic interactions with TYR61 were also observed. Most of the tested compounds showed comparable interactions with the surrounding amino acid residues, with a comparable binding mode (Table 3, Figure 2).

### 2.4. Structure–Activity Relationship

**Figure 3:**
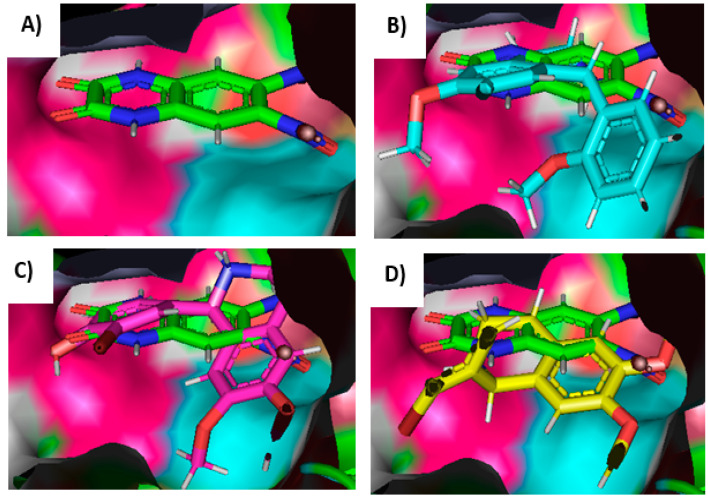
3D interaction with amino acid residues in the active site of (**A**): ligand, (**B**): overlay of ligand and compound **6** (cyan), (**C**): overlay of ligand and compound **18** (magenta), (**D**): overlay of ligand and compound **27** (yellow). Residues: PRO89, THR91, and ARG96 pink, GLU193 cyan, LYS218 yellow, MET196 orange, visualized by Paymol.

Molecular docking results were analyzed to study the essential pharmacophoric features in the studied scaffold represented as (1-aryl-6,7-dimethoxy-1,2,3,4-tetrahydroisoquinoline-1-ArTHIQ) derivatives. Compound **18** showed the best conformation with the most promising binding pose in the AMPA binding pocket. Results were superior regarding both the binding interactions with the key amino acid residues and the C-Docker binding interaction energy (E = −45.41 Kcal/mol), confirming good fitting with stable binding interaction exceeding the ligand, where three conventional hydrogen bonds were assured within the active site, and two carbon hydrogen bonds (Table 3). It is worth noting that hydrogen bonds play a crucial role in protein–ligand interactions [20,21]. The presence of the 4′-hydroxyphenyl group allows two hydrogen bindings, in addition to the hydrophobic halogen bond with 3′-bromo- substitution. The hydrophobic interaction is known, together with hydrogen bonds, to stabilize ligands at the receptor site [20,22,23]. On the other side, compound **13** showed the least binding profile compared to the ligand (E = −33.82 Kcal/mol), showing only one carbon hydrogen interaction apart from the hydrophobic binding and interaction with 2′-bromo substitution in the phenyl group. Although the 4′-hydroxyphenyl was essential, as in compounds **4** and **6**, and the 4′-methoxyphenyl group showed important binding, as in compounds **5** and **27**, it was observed that the replacement of the 4′-hydroxy-3′-methoxyphenyl substituent in the phenyl group by cyclization to 4′,5′-methylenedioxy, as in compounds **29**, **30**, and **31**, retained the essential interactions needed for biological activity. Additionally, the 2′-chlorophenyl group in compound **31** showed better results than the 2′-bromo substitution as in compound **13**. In compounds **23** and **26**, the addition of 2′-nitrophenyl in the presence of 3′-methoxy or 4′,5′-dimethoxy enhanced the binding for both compounds within the active site, in contrast to the 2′-nitrophenyl in compounds **7**, **8**, and **9** lacking the methoxy groups (Figure 2). On the other side, the 2′, 3′, or 4′-aminophenyl position change showed no significance in compounds **32**, **33**, and **34** compared to the 4′-dimethylaminophenyl substitution in compound **16**. An overlay between the ligand and compounds **6**, **18**, and **27** at the active site was presented (Figure 3).

In summary, the visual analysis of the binding interactions of the studied compounds revealed the essential pharmacophoric features (Figure 2), represented in the existence of the 6,7-dimethoxy-1,2,3,4-tetrahydroisoquinoline moiety attached to 4′-hydroxyphenyl or 4′-methoxyphenyl with the 2′-chlorophenyl group better than the 2′-bromophenyl group. The replacement of the 4′-hydroxy-3′-methoxyphenyl substituent in the phenyl group by cyclization to 4′,5′-methylenedioxy was also considered. Also, 2′-nitrophenyl replacement with one or two methoxy substituents forming 3′-methoxy or 4′,5′-dimethoxy, fulfilled the required two hydrogen bonds, two hydrophobic bonds, and one ring aromatic pharmacophore [19].

### 2.5. Predictive ADMET Study

**Figure 4:**
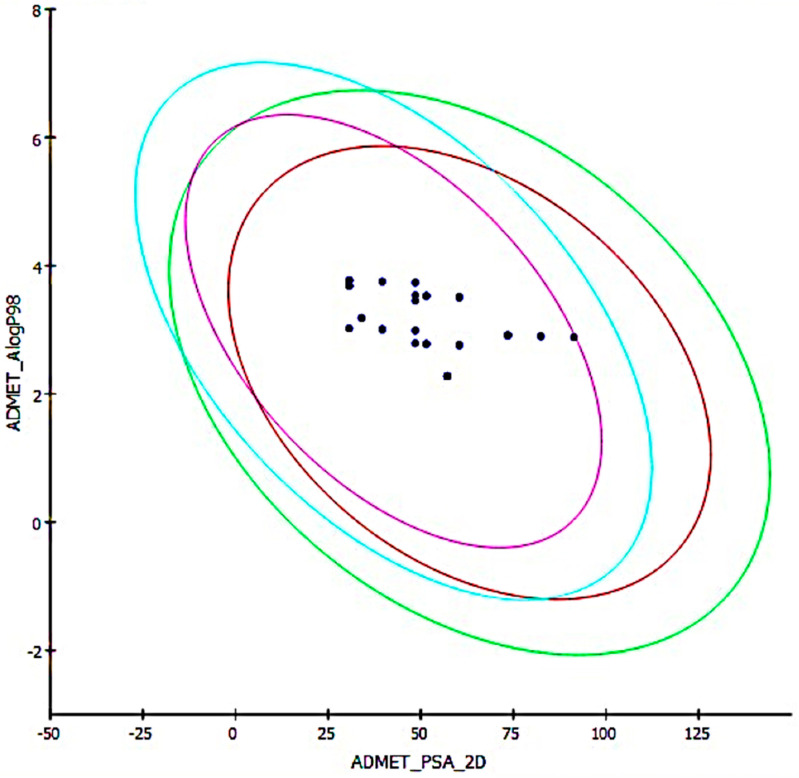
ADMET plot of the tested compounds: calculated PSA_2D versus A log P98 properties.

A predictive ADMET study was conducted via Discovery Studio 4.0 software on the tested compounds to determine their pharmacokinetic profile and investigate their drug-likeness. Results based on the chemical structure of the compounds, presented in Table 4, were greatly correlated to different parameters, such as aqueous solubility level, absorption level, blood–brain barrier level, hepatotoxicity probability, atom-based log P98 (A LogP 98), plasma protein binding level (PPB Level), cytochrome P450 2D6 (CYP2D6), and 2D polar surface area (ADMET 2D PSA) (Figure 4).

The results demonstrated in Table 4 showed an absorption level range of (0) for all the compounds, indicating a good level of human intestinal absorption. The compounds revealed ADME aqueous solubility level values of (2–3), confirming good aqueous solubility. Only compounds **23** and **26** exerted a BBB penetration level of 3, indicating that they could not pass the BBB, while all the other compounds lay between high values (1) and medium (2) BBB penetration levels. The log P parameter is used to detect the compound lipophilicity, being evaluated with the polar surface area (PSA) parameter, where compounds with values lower than 5 usually showed good absorption. The PSA property is considered an important key to determine the bioavailability of potential drugs. Molecules with PSA < 140 showed passive absorption. Thus, the tested compounds were predicted to show good absorption, with probability of hepatotoxicity. The compounds were also determined to be non-inhibitors to cytochrome P450 2D6 (CYP2D6), with probability of plasma protein binding (Table 4). The TopKat toxicity studies showed that all properties and OPS (operating properties of systems) components were within expected ranges. The carcinogenic potency TD50 range was 0.678266–17.9936 mg/kg body weight/day, and all the tested molecules were non-carcinogens.

In the strychnine-induced seizures model, all studied 1-ArTHIQ reduced animal mortality by 20–90%, depending on the administered dose, and compounds **20** and **25** were the most active. The analysis of the structure–activity relationship showed that for the highest survival rate of strychnine-poisoned animals (90%), the simultaneous presence of a bromine atom at C-2′ and methoxy groups at C-4′ and C-5′ is necessary (compound **25**). A similar effect was observed when a hydroxyl group was present at C-3′ alongside a methoxy group at C-4′ (compound **20**). When studying the effects of 1-ArTHIQ and reference drugs on the tremorogenic and convulsive actions of nicotine at doses of 0.1–5 mg/kg, the compounds **3**, **6**, **8**, **14**, **16**, **25**, **27**, **29**, **30**, **31,** and **34** showed comparable activity to the reference drugs. These compounds, at low doses, increased the survival rate of the animals to 90–100%. The results indicate that compounds **27**, with an LD_50_ of 4700 mg/kg, and **18**, with an LD_50_ of 3850 mg/kg, exhibit the lowest toxicity combined with high anticonvulsant activity in the nicotine seizure model. These compounds possess both a bromine atom and a hydroxyl group at C-2′ and C-3′, contributing to a survival rate of 90% in animals starting from a dose of 0.5 mg/kg. Compound **6** demonstrates the highest anticonvulsant activity, leading to a survival rate of 90% in nicotine-poisoned animals from a dose of 0.1 mg/kg. The data from this study indicate that the anticonvulsant effects of leading substances from the examined series of 1-ArTHIQ may be related to their action on glycine and α7 nACh receptors. Some of the studied compounds may serve as lead compounds for the development of new anticonvulsant medications. The in silico results showed promising docking interaction of most of the tested compounds. The prediction of the structural requirements necessary for the observed anticonvulsant activity was explained via molecular simulation study and showed potential results of the studied scaffold. Furthermore, the pharmacokinetic properties and drug-likeness of the tested compounds were investigated, and they showed good profiles as potential anticonvulsant agents. In addition, the toxicity testing generated by Discovery Studio 4.0 confirmed the non-mutagen results of all the compounds.

## 3. Discussion

The analysis of the structure–activity relationship showed that for the highest survival rate of strychnine-poisoned animals (90%), the simultaneous presence of a bromine atom at C-2′ and methoxy groups at C-4′ and C-5′ is necessary (compound **25**). A similar effect was observed when a hydroxyl group was present at C-3′ alongside a methoxy group at C-4′ (compound **20**). The presence of methoxy groups at C-3′ and C-4′ (compound **22**) and C-2′ (compound **6**), along with a bromine atom at either C-2′ (compound **13**) or C-4′ (compound **15**), resulted in a survival rate of no more than 40% in poisoned animals. In contrast, a methoxy group at C-4′ (compound **5**) and a bromine atom at C-3′ (compound **14**) led to a survival rate of up to 60%. The presence of hydroxyl groups at C-2′ (compound **3**) and C-3′ (compound **4**) also resulted in a survival rate of up to 60% in the animals.

The action of strychnine is associated with the facilitation of excitation transmission at the inter-neuronal synapses in the spinal cord, primarily affecting the interneurons. According to current understanding, strychnine blocks the postsynaptic action of glycine, which serves as an inhibitory factor in the transmission of excitation at postsynaptic nerve endings in the spinal cord [24].

In the evaluation of the effects of the studied compounds at doses of 0.1–5 mg/kg and reference drugs on nicotine-induced tremors and convulsions, the compounds **3**, **6**, **8**, **14**, **16**, **25**, **27**, **29**, **30**, **31,** and **34** showed comparable activity to the reference drugs. These compounds, at low doses, increased the survival rate of the animals to 90–100%. The results indicate that compounds **27**, with an LD_50_ of 4700 mg/kg, and **18**, with an LD_50_ of 3850 mg/kg, exhibit the lowest toxicity combined with high anticonvulsant activity in the nicotine seizure model. These compounds possess both a bromine atom and a hydroxyl group at C-2′ and C-3′, contributing to a survival rate of 90% in animals starting from a dose of 0.5 mg/kg. Compound **6** demonstrates the highest anticonvulsant activity, leading to a survival rate of 90% in nicotine-poisoned animals from a dose of 0.1 mg/kg. The simultaneous presence of a bromine atom, methoxy, and hydroxyl groups as substituents in various combinations (compounds **24**, **27**, **28**) did not improve the survival of poisoned animals compared to carbamazepine and valproate.

Among the studied derivatives, the most toxic substances are **2–34** without a substituent in the aromatic ring at position C (**2**) or containing a single substituent (OH, OCH_3_, Br, Cl) in the *ortho*- or *meta*-position (**3**, **6**, **10**, **11**, **13**, **14**), as well as the methylenedioxy group (**29**). These substituents in the para-position usually reduce toxicity (**4**, **5**, **9**). Introducing two substituents in the meta- and para-positions decreases toxicity, which also depends on the nature and mutual arrangement of these groups. For example, toxicity decreases in the series **22** > **20** > **21** (OH, OCH_3_), **19** (LD_50_ 327.9 mg/kg) >> **18** (LD_50_ 3793.8 mg/kg) > **8** (OH or OCH_3_, and Br). The trisubstituted derivative **27** was less toxic among the derivatives **2**–**34** (LD_50_ 4937 mg/kg).

Nicotine induces convulsive seizures by activating amygdalar neurons primarily through α7 nACh receptors [25]. The data from this study indicate that the anticonvulsant effects of leading substances from the examined series of 1-aryl-1,2,3,4-tetrahydroisoquinolines may be related to their action on glycine and α7 nACh receptors. Some of the studied compounds may serve as lead compounds for the development of new anticonvulsant medications. The in silico results showed promising docking interaction of most of the tested compounds. The prediction of the structural requirements necessary for the observed anticonvulsant activity was explained via molecular simulation study and showed potential results of the studied scaffold.

## 4. Materials and Methods

### 4.1. Synthesis of the Compounds

Derivatives of 1-aryl-1,2,3,4-tetrahydroisoquinoline **2**–**31** were obtained as described in [18].

### 4.2. Biological Evaluation

The anticonvulsant effects of the examined substances were studied in male white mice weighing 18–22 g, divided into the following groups: control (including the strychnine and nicotine groups), carbamazepine group, Convulex group, and experimental groups (one for each tested substance). Simple randomization (“heads or tails” coin toss) was used to assign animals to groups. Each group contained 10 animals. Healthy animals were included in the experiments after a 14-day quarantine period. No animals were excluded from the study. Convulsant agents were administered subcutaneously at doses of 10.0 mg/kg for nicotine (Sigma–Aldrich, Saint Louis, MO, USA) and 1.5 mg/kg for strychnine (Sigma–Aldrich, Saint Louis, MO, USA), 60 min after the oral administration of the test substances at doses ranging from 0.1 to 10 mg/kg. The onset time, duration of tremors and seizures, and survival rate of the animals were recorded. Carbamazepine (Darnitsa, Kiev, Ukraine) at doses of 5.0–10.0 mg/kg and Convulex (valproate) (Gerot Pharmazeutika GmbH, Vienna, Austria) at doses of 75.0–100.0 mg/kg (based on the therapeutic anticonvulsant dose) were used as reference drugs.

Data were presented as mean ± SD for different groups. Statistical analysis was performed using OriginPro 9.0 (MicroCal Software, Northampton, MA, USA), with *p* < 0.05 considered statistically significant.

### 4.3. Molecular Modeling

The crystal structures of the GluR2 ligand binding core (S1S2), in complex with the antagonist (6,7-dinitroquinoxaline-2,3-dione) (PDB ID: 1FTL), were downloaded from Protein Data Bank [26] to investigate the antagonistic mechanism against the AMPA-sensitive glutamate receptor. The molecular docking study was applied using Discovery Studio 4.0 software using the C-Docker algorithm. Protein preparation was confirmed by Clean Protein, to check for missing residues completion and water molecules or any unnecessary radicals’ removal. CHARMM Force Field simulation was applied, and protein was minimized. The active site was identified by selecting the ligand before removal for re-docking with the prepared ligands composed of 32 tested compounds. Visual inspection of the docking results was further carried out, together with C-Docker interaction energy (E) inspection using Discovery Studio 4.0 software [27], to investigate the binding mode of the tested compounds compared to that of the ligand and reported literature [20]. The best pose out of ten per each compound was selected, and the binding mode with the essential amino acid residues within the binding site was represented showing different binding interactions, including hydrogen bonds or carbon–hydrogen bonds, hydrophobic bonds as pi–pi or pi–alkyl, and Van der Waals interactions [27]. A predictive in silico ADMET study using Discovery Studio 4.0 software was conducted to evaluate the pharmacokinetic profile and the drug-likeness of all tested compounds and to record any predicted toxicity [21].

## Figures and Tables

**Table 1:** Anticonvulsant effects of examined substances in the strychnine-induced seizures model.

№	Tested Compound	Dose (mg/kg)	Latency to Tremor and Seizure Onset (min)	Duration of Tremor and Seizures (min)	Survival Rate (%)
**2**	1-Phenyl-6,7-dimethoxy-1,2,3,4-tetrahydroisoquinoline	1.0	14.1 ± 1.5	0.43 ± 0.9	60
**3**	1-(2′-Hydroxyphenyl)-6,7-dimethoxy-1,2,3,4-tetrahydroisoquinoline	1.0	14.5 ± 1.7	0.29 ± 0.6	60
**4**	1-(4′-Hydroxyphenyl)-6,7-dimethoxy-1,2,3,4-tetrahydroisoquinoline	1.0	14.2 ± 1.8	0.57 ± 0.7	70
**5**	1-(4′-Methoxyphenyl)-6,7-dimethoxy-1,2,3,4-tetrahydroisoquinoline	1.0	16.5 ± 1.8	0.54 ± 0.7	60
**6**	1-(2′-Methoxyphenyl)-6,7-dimethoxy-1,2,3,4-tetrahydroisoquinoline	1.0	15.7 ± 1.7	0.50 ± 0.4	30
**7**	1-(2′-Nitrophenyl)-6,7-dimethoxy-1,2,3,4-tetrahydroisoquinoline	1.0	14.2 ± 1.7	0.32 ± 0.7	40
**8**	1-(3′-Nitrophenyl)-6,7-dimethoxy-1,2,3,4-tetrahydroisoquinoline	1.0	15.9 ± 1.7	0.56 ± 0.4	40
**9**	1-(4′-Nitrophenyl)-6,7-dimethoxy-1,2,3,4-tetrahydroisoquinoline	1.0	13.5 ± 1.9	0.51 ± 0.5	40
**10**	1-(2′-Chlorophenyl)-6,7-dimethoxy-1,2,3,4-tetrahydroisoquinoline	1.0	17.2 ± 1.7	0.60 ± 0.4	30
**11**	1-(3′-Chlorophenyl)-6,7-dimethoxy-1,2,3,4-tetrahydroisoquinoline	1.0	15.5 ± 1.2	0.46 ± 0.5	70
**12**	1-(4′-Chlorophenyl)-6,7-dimethoxy-1,2,3,4-tetrahydroisoquinoline	1.0	18.5 ± 1.8	0.23 ± 0.6	20
**13**	1-(2′-Bromophenyl)-6,7-dimethoxy-1,2,3,4-tetrahydroisoquinoline	1.0	10.5 ± 1.3	0.31 ± 0.7	40
**14**	1-(3′-Bromophenyl)-6,7-dimethoxy-1,2,3,4-tetrahydroisoquinoline	1.0	18.5 ± 1.5	0.35 ± 0.8	60
**15**	1-(4′-Bromophenyl)-6,7-dimethoxy-1,2,3,4-tetrahydroisoquinoline	1.0	12.5 ± 1.2	0.50 ± 0.5	40
**16**	1-(4′-Dimethylaminophenyl)-6,7-dimethoxy-1,2,3,4-tetrahydroisoquinoline	1.0	13.1 ± 1.5	0.31 ± 0.6	50
**17**	1-(2′-Hydroxy-5′-bromophenyl)-6,7-dimethoxy-1,2,3,4-tetrahydroisoquinoline	1.0	15.7 ± 1.6	0.61 ± 0.5	40
**18**	1-(3′-Bromo-4′-hydroxyphenyl)-6,7-dimethoxy-1,2,3,4-tetrahydroisoquinoline	1.0	14.6 ± 1.5	0.56 ± 0.6	50
**19**	1-(3′-Bromo-4′-methoxyphenyl)-6,7-dimethoxy-1,2,3,4-tetrahydroisoquinoline	1.0	16.0 ± 1.4	0.59 ± 0.9	40
**20**	1-(3′-Hydroxy-4′-methoxyphenyl)-6,7-dimethoxy-1,2,3,4-tetrahydroisoquinoline	1.0	18.2 ± 1.4	0.63 ± 0.7	90
**21**	1-(4′-Hydroxy-3′-methoxyphenyl)-6,7-dimethoxy-1,2,3,4-tetrahydroisoquinoline	1.0	15.4 ± 1.5	0.52 ± 0.4	40
**22**	1-(3′,4′-Dimethoxyphenyl)-6,7-dimethoxy-1,2,3,4-tetrahydroisoquinoline	1.0	12.7 ± 1.3	0.71 ± 0.6	30
**23**	1-(3′-Methoxy-2′-nitrophenyl)-6,7-dimethoxy-1,2,3,4-tetrahydroisoquinoline	1.0	14.6 ± 1.4	0.60 ± 0.4	30
**24**	1-(2′-Bromo-4′-methoxy-5′-hydroxyphenyl)-6,7-dimethoxy-1,2,3,4-tetrahydroisoquinoline	1.0	10.2 ± 1.2	0.32 ± 0.4	30
**25**	1-(2′-Bromo-4′,5′-dimethoxyphenyl)-6,7-dimethoxy-1,2,3,4-tetrahydroisoquinoline	1.0	16.5 ± 1.5	0.14 ± 0.6	90
**26**	1-(4′,5′-Dimethoxy-2′-nitrophenyl)-6,7-dimethoxy-1,2,3,4- tetrahydroisoquinoline	1.0	16.7 ± 1.2	0.51 ± 0.8	40
**27**	1-(2′-Bromo-3′-hydroxy-4′-methoxyphenyl)-6,7-dimethoxy-1,2,3,4-tetrahydroisoquinoline	1.0	10.5 ± 1.5	0.53 ± 0.3	40
**28**	1-(5′-Bromo-4′-hydroxy-3′-methoxyphenyl)-6,7- dimethoxy-1,2,3,4-tetrahydroisoquinoline	1.0	15.5 ± 1.5	0.34 ± 0.8	20
**29**	1-(3′,4′-Methylenedioxyphenyl)-6,7-dimethoxy-1,2,3,4-tetrahydroisoquinoline	1.0	12.5 ± 1.8	0.32 ± 0.9	50
**30**	1-(2′-Bromo-4′,5′-methylenedioxyphenyl)-6,7-dimethoxy-1,2,3,4-tetrahydroisoquinoline	1.0	11.2 ± 1.2	0.41 ± 0.4	30
**31**	1-(4′,5′-Methylenedioxy-2′-chlorophenyl)-6,7-dimethoxy-1,2,3,4-tetrahydroisoquinoline	1.0	13.6 ± 1.9	0.59 ± 0.5	30
**32**	1-(2′-Aminophenyl)-6,7-dimethoxy-1,2,3,4-tetrahydroisoquinoline	1.0	18.2 ± 1.7	0.34 ± 0.4	50
**33**	1-(3′-Aminophenyl)-6,7-dimethoxy-1,2,3,4-tetrahydroisoquinoline	1.0	16.7 ± 1.0	0.42 ± 0.8	50
**34**	1-(4′-Aminophenyl)-6,7-dimethoxy-1,2,3,4-tetrahydroisoquinoline	1.0	15.5 ± 1.8	0.34 ± 0.7	40
**35**	Control (Strychnine)	1.5	9.8 ± 1.2	0.52 ± 0.7	0
**36**	Carbamazepine	5.0	14.6 ± 1.4	0.43 ± 0.5	40
		10.0	12.6 ± 1.7	0.38 ± 0.8	50
**37**	Valproate (Convulex)	75.0	15.4 ± 1.5	0.34 ± 0.6	40
		100.0	14.3 ± 1.3	0.32 ± 0.4	20

Data are shown as mean ± SD of different groups (N = 10). A *p* < 0.05 was considered statistically significant.

**Table 2:** Effects of examined substances on tremorogenic and convulsive actions of nicotine.

Compound	Dose (mg/kg)	LD50 (mg/kg, per os)	Latency to Seizure Onset (min)	Duration of Seizures (min)	Survival Rate (%)	Compound	Dose (mg/kg)	LD50 (mg/kg, per os)	Latency to Seizure Onset (min)	Duration of Seizures (min)	Survival Rate (%)
**2**	1.0	273.2 (249.7 ± 296.7)	3.2 ± 0.07	3.2 ± 0.6	70	**1** **9**	1.0	327.9 (287.1 ± 363.8)	1.8 ± 0.07	19.0 ± 0.8	80
**3**	1.0	365.3 (326.1 ± 394.5)	2.6 ± 0.08	9.0 ± 0.4	90	20	1.0	964.8 (916.5 ± 998.2)	2.8 ± 0.07	11.0 ± 0.7	70
**4**	1.0	924.2 (893.9 ± 953.9)	1.8 ± 0.05	10.5 ± 0.8	60	21	1.0	1663.6 (1623.1 ± 1629.1)	1.8 ± 0.04	14.3 ± 0.6	60
**5**	1.0	1040.3 (986.0 ± 1079.8)	2.1 ± 0.09	10.3 ± 0.6	80	22	1.0	666.6 (557.9 ± 760.2)	2.2 ± 0.04	8.4 ± 0.50.5	80
**6**	1.0	286.7 (256.9 ± 318.7)	2.4 ± 0.03	10.5 ± 0.5	100	23	1.0	315.4 (281.0 ± 344.2)	1.5 ± 0.08	5.4 ± 0.9	20
**7**	1.0	948.9 (916.8 ± 975.8)	2.3 ± 0.07	7.6 ± 0.8	70	24	1.0	546.9 (516.3 ± 571.6)	2.1 ± 0.09	7.3 ± 0.3	70
**8**	1.0	531.4 (501.3 ± 554.1)	2.3 ± 0.08	15.5 ± 0.3	90	25	1.0	272.1 (249.1 ± 295.6)	2.2 ± 0.04	9.0 ± 0.9	90
**9**	1.0	1898.8 (703.5 ± 2273.4)	2.5 ± 0.03	16.7 ± 0.4	90	26	1.0	524.5 (501.3 ± 547.7)	2.6 ± 0.03	10.8 ± 0.4	80
**10**	1.0	264.2 (224.8 ± 293.9)	2.2 ± 0.03	9.2 ± 0.9	70	27	1.0	4937.4 (4827.7 ± 5084.9)	2.2 ± 0.02	9.7 ± 0.9	90
**11**	1.0	232.4 (211.8 ± 251.1)	2.2 ± 0.07	8.3 ± 0.3	70	28	1.0	1532.7 (1471.5 ± 1579.5)	2.7 ± 0.04	9.2 ± 0.4	70
**12**	1.0	730.4 (598.2 ± 883.7)	2.1 ± 0.09	7.0 ± 0.4	70	29	1.0	267.2 (226.3 ± 301.1)	2.5 ± 0.07	3.7 ± 0.9	90
**1** **3**	1.0	315.8 (281.2 ± 345.2)	2.2 ± 0.03	5.0 ± 0.7	50	30	1.0	711.7 (604.3 ± 792.9)	2.5 ± 0.09	7.0 ± 0.2	90
**1** **4**	1.0	446.7 (416.2 ± 471.3)	2.8 ± 0.02	2.5 ± 0.4	90	31	1.0	520.8 (491.9 ± 545.4)	2.3 ± 0.08	10.7 ± 0.9	90
**1** **5**	1.0	422.0 (399.4 ± 445.3)	2.2 ± 0.07	8.3 ± 0.3	70	32	1.0	366.3 (326.3 ± 395.6)	1.8 ± 0.06	12.2 ± 0.8	60
**1** **6**	1.0	498.3 (471.6 ± 524.6)	2.7 ± 0.08	8.5 ± 0.7	90	33	1.0	732.0 (598.2 ± 885.4)	2.2 ± 0.04	17.0 ± 0.6	80
**17**	1.0	297.3 (270.2 ± 323.7)	2.1 ± 0.07	8.1 ± 0.8	70	34	1.0	1043.2 (993.3 ± 1082.6)	2.2 ± 0.04	10.2 ± 0.7	90
**18**	1.0	3793.8 (3395.8 ± 4088.1)	1.7 ± 0.04	8.9 ± 0.9	70	Nicotine	10.0		40^sec^ ± 0.03	13.5 ± 0.8	30
						Carbamazepine	5.0		1.57 ± 0.07	15.2 ± 0.9	60
							10.0		1.5 ± 0.02	4.7 ± 0.6	80
						Valproate	75.0		1.2 ± 0.04	6.2 ± 0.4	90
							100.0		1.5 ± 0.06	10.8 ± 0.8	90

Data are shown as mean ± SD of different groups (N = 10). A *p* < 0.05 was considered statistically significant. sec = seconds.

**Table 3:** Docking score (kcal/mol) and interactions of compounds **2–34** in the active site of AMPA (PDB ID: 1FTL).

Compound	Ligand/C-Docker Interaction Energy (Kcal/mol)	Amino Acid Residues Interaction
Ligand (6,7-dinitroquinoxaline-2,3-dione)	−41.60	2 HBAs with **ARG96**, 1 HBA with **THR195**, **GLU193**, and **THR174,** 1 HBD with **PRO89,** 2 pi–pi bonds with **TYR 61,** 1 carbon–hydrogen bond with **LEU90**
**2**	−34.78	1 HBD and 1 carbon–hydrogen bond with **THR91**, 1 carbon–hydrogen bond with **THR143**, 1 pi–pi bond and 1 pi–carbon with **TYR 61,** 1 pi–alkyl with **LEU138**
**3**	−39.20	2 HBAs with **ARG96**, 1 HBD with **THR174,** 1 carbon–hydrogen bond with **GLY62,** 2 pi–pi bonds with **TYR 61,** 1 pi–alkyl with **MET196,** 1 donor–donor with **GLU193**
**4**	−36.33	2 HBAs with **ARG96**, 1 carbon–hydrogen bond with **GLY62,** 2 pi–pi bonds with **TYR 61,** 1 donor–donor with **GLU193**
**5**	−38.24	1 HBD and 1 carbon–hydrogen bond with **THR91**, 1 carbon–hydrogen bond with **THR143**, 1 carbon–hydrogen bond with **SER140**, 1 pi–pi bond and 1 pi–carbon with **TYR 61,** 1 pi–alkyl with **LEU138**
**6**	−37.21	1 HBA with **ARG96**, 1 carbon–hydrogen bond with **THR91,** 2 carbon–hydrogen bonds with **GLU193,** 1 pi–pi bond with **TYR 61,** 1 pi–sigma with **LEU138**
**7**	−35.31	1 HBA with **ARG96**, **THR91**, and **SER142,** 2 pi–pi bonds with **TYR 61,** 1 carbon–hydrogen bond with **PRO89 and TYR220,** 1 attractive charge with **LYS218**
**8**	−38.23	1 HBA with **ARG96**, 1 carbon–hydrogen bond with **THR143,** 2 pi–pi bonds with **TYR 61,** 1 pi–sigma with **LEU138,** 1 donor–donor with **GLU193** and **TYR220**
**9**	−38.09	1 HBD and 1 carbon–hydrogen bond with **THR91**, 1 carbon–hydrogen bond with **THR143**, 1 pi–pi bond and 1 pi–carbon with **TYR 61,** 1 pi–alkyl with **LEU138**
**10**	−34.46	1 HBD with **THR91**, 1 carbon–hydrogen bond with **THR143** and **SER142 (halogen bond)**, 1 pi–pi bond and 1 pi–carbon with **TYR 61,** 2 pi–alkyl with **LEU138**
**11**	−36.48	2 carbon–hydrogen bonds with **THR91,** 1 carbon–hydrogen bond with **PRO89,** 1 pi–pi bond and 1 pi–carbon with **TYR 61** and 1 alkyl bond **(halogen bond),** 1 pi–alkyl with **LEU138** and 1 alkyl bond **(halogen bond)**
**12**	−36.62	1 HBD and 1 carbon–hydrogen bond with **THR91**, 1 carbon–hydrogen bond with **THR143**, 1 pi–pi bond and 1 pi–carbon with **TYR 61,** 1 alkyl bond **(halogen bond)** with **ALA63**
**13**	−33.82	1 carbon–hydrogen bond with **PRO89** and **THR143,** 1 pi–pi bond with **TYR 61** and 1 alkyl bond **(halogen bond)**
**14**	−38.00	1 carbon–hydrogen bond with **PRO89** and **THR143,** 2 carbon–hydrogen bonds with **THR91,** 1 pi–pi bond and 1 alkyl bond **(halogen bond)** with **TYR 61,** 1 pi–alkyl with **LEU138** and 1 alkyl bond **(halogen bond)**
**15**	−35.83	2 HBDs with **THR91**, 1 HBA with **THR174** and 1 alkyl bond with **ALA63 (halogen bond)**, 1 pi–carbon with **GLU193,** 1 pi–alkyl with **LEU138**
**16**	−41.44	1 HBD with **THR91,** 1 carbon–hydrogen bond with **THR91, PRO89, THR143, and SER140,** 1 pi–carbon bond with **TYR 61**
**17**	−38.28	**Halogen bonds** with each of **TYR16, PRO89, and TYR220,** 1 carbon–hydrogen bond with **GLY62,** 1 donor–donor with **GLU193**
**18**	−45.41	1 HBA with **ARG96** and **THR91,** 1 HBD, 1 pi–pi bond, and 1 alkyl bond **(halogen bond)** with **TYR 61,** 1 carbon–hydrogen bond with **THR143,** 2 with **GLU193,** 1 pi–alkyl with **LEU138**
**19**	−39.87	1 HBD with **THR91**, 1 carbon–hydrogen bond with **THR143**, 1 pi–pi bond, 1 pi–carbon, and 1 alkyl bond **(halogen bond)** with **TYR 61,** 1 pi–alkyl, and 1 alkyl bond **(halogen bond)** with **LEU138**
**20**	−38.90	1 HBA with **ARG96,** 1 HBD with **GLU13 and TYR61,** 1 pi–pi bond with **TYR 61,** 1 carbon–hydrogen bond with **PRO89and THR91,** 1 pi–alkyl with **LEU138 and MET196**
**21**	−38.50	1 HBA with **ARG96,** 1 HBD, 1 attractive charge, and 1 donor–donor with **GLU193,** 1 carbon–hydrogen bond with **GLY62, PRO89, THR143, and TYR220,** 1 pi–pi bond with **TYR 61,** 1 pi–alkyl with **LEU138**
**22**	−41.19	1 HBD and 1 carbon–hydrogen bond with **THR91**, 1 carbon–hydrogen bond with **THR143** and **SER140,** 1 pi–pi bond and 1 pi–carbon with **TYR 61,** 1 pi–alkyl with **LEU138**
**23**	−44.10	3 HBAs with each of **ARG96, THR91, and SER142,** 1 pi–pi bond and 1 pi–carbon with **TYR 61,** 1 attractive charge with **LYS218,** 1 carbon–hydrogen bond with **THR143 and PRO89,** 2 carbon–hydrogen bonds with **THR91**
**24**	−41.48	1 HBA with **ARG96,** 1 HBD and 1 attractive charge with **GLU193,** 1 alkyl bond **(halogen bond)** with **TYR 61,** 1 carbon–hydrogen bond with **GLY62 and THR91**
**25**	−43.00	1 HBA with **ARG96, THR91, and SER142,** 1 pi–pi bond, 1 carbon–hydrogen bond, and 1 alkyl bond **(halogen bond)** with **TYR 61,** 1 carbon–hydrogen bond with **THR91, SER140, GLY141, and GLY62,** 1 attractive charge with **GLU193**
**26**	−43.48	3 HBAs with each of **ARG96, THR91, and SER142,** 1 pi–pi bond and 1 pi–carbon with **TYR 61,** 1 pi–carbon with **LYS218,** 1 carbon–hydrogen bond with **THR143 and PRO89,** 2 carbon–hydrogen bonds with **THR91,** 1 attractive charge with **LYS218,** 1 pi–alkyl with **LEU138**
**27**	−38.67	1 HBD with **THR91,** 1 pi–pi bond and 1 pi–carbon with **TYR 61,** 2 carbon–hydrogen bonds with **SER140**, 1 with **THR143,** 2 pi–alkyl with **LEU138**
**28**	−39.89	1 HBA with **ARG96,** 2 pi–pi bonds with **TYR 61,** 1 carbon–hydrogen bond with **THR91 and GLY62,** 1 attractive charge with **GLU193, 3 halogen bonds** with each of **TYR 16, TYR220, and PRO89**
**29**	−42.46	1 HBD with **THR91,** 1 HBA with **SER142,** 1 pi–pi bond, 1 carbon–hydrogen bond, and 1 alkyl bond **(halogen bond)** with **TYR 61,** 1 carbon–hydrogen bond with **PRO89, LEU138, GLY141, and GLU145,** 1 pi–carbon with **TYR61**
**30**	−38.17	1 HBA with **ARG96,** 1 pi–pi bond with **TYR 61,** 1 carbon–hydrogen bond with **GLY62**, **PRO89, and GLU193,** 1 alkyl bond **(halogen bond)** with **LEU138 and LEU192**
**31**	−40.36	1 HBA with **SER142,** 1 HBD, 1 attractive charge, and 1 carbon–hydrogen bond with **GLU193,** 1 carbon–hydrogen bond with **TYR61, PRO89, THR91, and SER140,** 1 alkyl bond **(halogen bond)** with **TYR61**
**32**	−37.62	2 HBAs with **ARG96,** 1 HBD with **THR174,** 2 pi–pi bonds with **TYR 61,** 1 carbon–hydrogen bond with **GLY62,** 1 pi–alkyl with **MET196**
**33**	−37.06	2 carbon–hydrogen bonds with **THR91,** 1 carbon–hydrogen bond with **PRO89** and **THR143** and 2 with **THR91,** 1 pi–pi bond and 1 pi–carbon with **TYR61,** 1 pi–alkyl with **LEU138**
**34**	−34.41	1 HBD with **PRO89,** 1 donor–donor, 1 attractive charge, and 1 carbon–hydrogen bond with **GLU193,** 1 carbon–hydrogen bond with **SER140**

**Table 4:** The predictive in silico ADMET results of the tested compounds (**2–34**).

Compound	ADMET Sol Level	ADMET A Log P98	ADMET BBB Level	CYP2D6 Prediction (Non-Inhibitor)	Hepatotoxic Probability	Absorption Level	PPB Binding Prediction	Alog P98	PSA 2D	TOPKAT Carcinogenic Potency TD 50 Rat	TOPKAT Ames Prediction
**2**	2	0	1	FALSE	TRUE	0 (good)	TRUE	3.021	30.67	21.9697	Non-Mutagen
**3**	**3**	0	2	FALSE	TRUE	0 (good)	TRUE	2.779	51.485	49.3691	Non-Mutagen
**4**	**3**	0	2	FALSE	TRUE	0 (good)	TRUE	2.779	51.485	17.9936	Non-Mutagen
**5**	2	0	1	FALSE	TRUE	0 (good)	TRUE	3.005	39.6	5.46952	Non-Mutagen
**6**	2	0	1	FALSE	TRUE	0 (good)	TRUE	3.005	39.6	8.99628	Non-Mutagen
**7**	2	0	2	FALSE	TRUE	0 (good)	TRUE	2.916	73.493	4.87626	Non-Mutagen
**8**	2	0	2	FALSE	TRUE	0 (good)	TRUE	2.916	73.493	4.69132	Non-Mutagen
**9**	2	0	2	FALSE	TRUE	0 (good)	TRUE	2.916	73.493	1.77725	Non-Mutagen
**10**	2	0	1	FALSE	TRUE	0 (good)	TRUE	3.686	30.67	6.10272	Non-Mutagen
**11**	2	0	1	FALSE	TRUE	0 (good)	TRUE	3.686	30.67	5.87126	Non-Mutagen
**12**	2	0	1	FALSE	TRUE	0 (good)	TRUE	3.686	30.67	2.22426	Non-Mutagen
**13**	2	0	1	FALSE	TRUE	0 (good)	TRUE	3.77	30.67	6.51486	Non-Mutagen
**14**	2	0	1	FALSE	TRUE	0 (good)	TRUE	3.77	30.67	6.26777	Non-Mutagen
**15**	2	0	1	FALSE	TRUE	0 (good)	TRUE	3.77	30.67	2.37448	Non-Mutagen
**16**	2	0	1	FALSE	TRUE	0 (good)	TRUE	3.184	34.022	7.61886	Non-Mutagen
**17**	2	0	1	FALSE	TRUE	0 (good)	TRUE	3.528	51.485	9.89607	Non-Mutagen
**18**	2	0	1	FALSE	TRUE	0 (good)	TRUE	3.528	51.485	12.5742	Non-Mutagen
**19**	2	0	1	FALSE	TRUE	0 (good)	TRUE	3.753	39.6	3.98643	Non-Mutagen
**20**	**3**	0	2	FALSE	TRUE	0 (good)	TRUE	2.763	60.416	22.3668	Non-Mutagen
**21**	**3**	0	2	FALSE	TRUE	0 (good)	TRUE	2.763	60.416	17.9743	Non-Mutagen
**22**	2	0	1	FALSE	TRUE	0 (good)	TRUE	2.989	48.53	5.73009	Non-Mutagen
**23**	2	0	**3**	FALSE	TRUE	0 (good)	TRUE	2.899	82.423	7.94352	Non-Mutagen
**24**	2	0	2	FALSE	TRUE	0 (good)	TRUE	3.511	60.416	7.04772	Non-Mutagen
**25**	2	0	1	FALSE	TRUE	0 (good)	TRUE	3.737	48.53	1.79015	Non-Mutagen
**26**	2	0	**3**	FALSE	TRUE	0 (good)	TRUE	2.883	91.353	1.36115	Non-Mutagen
**27**	2	0	2	FALSE	TRUE	0 (good)	TRUE	3.511	60.416	15.3108	Non-Mutagen
**28**	2	0	2	FALSE	TRUE	0 (good)	TRUE	3.511	60.416	7.04772	Non-Mutagen
**29**	2	0	2	FALSE	TRUE	0 (good)	TRUE	2.789	48.53	2.25834	Non-Mutagen
**30**	2	0	1	FALSE	TRUE	0 (good)	TRUE	3.538	48.53	0.712376	Non-Mutagen
**31**	2	0	1	FALSE	TRUE	0 (good)	TRUE	3.454	48.53	0.678266	Non-Mutagen
**32**	**3**	0	2	FALSE	TRUE	0 (good)	TRUE	2.275	57.21	16.9758	Non-Mutagen
**33**	**3**	0	2	FALSE	TRUE	0 (good)	TRUE	2.275	57.21	6.88315	Non-Mutagen
**34**	**3**	0	2	FALSE	TRUE	0 (good)	TRUE	2.275	57.21	2.6076	Non-Mutagen

## Data Availability

The original contributions presented in this study are included in the article. Further inquiries can be directed to the corresponding authors.
